# Effects of Rhythmic Sensory Stimulation on Ehlers–Danlos Syndrome: A Pilot Study

**DOI:** 10.1155/2020/3586767

**Published:** 2020-04-27

**Authors:** Veronica Vuong, Abdullah Mosabbir, Denise Paneduro, Larry Picard, Hanna Faghfoury, Michael Evans, Allan Gordon, Lee Bartel

**Affiliations:** ^1^Faculty of Music, University of Toronto, Toronto, Ontario, Canada; ^2^Wasser Pain Management Centre, Sinai Health System, Toronto, Ontario, Canada; ^3^Division of Medical Oncology and Hematology, Sinai Health System, Toronto, Ontario, Canada; ^4^Department of Statistical Sciences, University of Toronto, Toronto, Ontario, Canada

## Abstract

Ehlers–Danlos syndrome (EDS) is a connective tissue disorder characterized by joint hypermobility and skin extensibility and is often accompanied by chronic pain. Rhythmic sensory stimulation (RSS) can be defined as the stimulation of the senses in a periodic manner within a range of low frequencies. Music plus sound delivered through a vibroacoustic device is a form of RSS and has demonstrated utility in managing pain. In this current study, we conducted an open-label pilot study of 15 patients with hypermobile EDS using RSS as the intervention. Posttreatment improvements were seen in 11 of the 15 patients (73%), whereas 3 of the 15 patients (20%) experienced worse outcomes. Of the 14 patients that completed the experiment, 6 participants (43%) were classified as “responders” to the device while 8 participants (57%) were classified as “nonresponders.” Responders demonstrated significant improvements in pain interference (51.5 ± 16 preintervention vs. 43.5 ± 16.4 postintervention BPI score) and depression symptoms (34.0 ± 15.9 preintervention vs. 26.8 ± 12.1 postintervention CESD score). Poststudy interviews confirm the improvements of pain interference, mood, and bowel symptoms. Furthermore, analysis of medical conditions within the responder group indicates that the presence of depression, anxiety, irritable bowel syndrome, and fibromyalgia may indicate a greater likelihood for patients to benefit with vibroacoustic applications. These results indicate a possible potential for RSS, delivered using a vibroacoustic device, in managing pain-related symptoms. Further research is necessary to elucidate the exact mechanism behind the physiological benefits of RSS.

## 1. Introduction

Ehlers–Danlos syndrome (hEDS) is a congenital, heterogeneous group of connective tissue disorders characterized by joint hypermobility and skin extensibility [1]. Many forms of hEDS are caused by genetic mutations coding for various types of fibrillar collagen leading to weak muscles, ligaments, tendons, and organs [2]. EDS is now classified under 13 different subtypes [3], with hypermobile EDS (hEDS) being the most common [1, 4]. Patients with hEDS may develop chronic pain that occurs due to multiple contributions from joint wear and tear, musculoskeletal stiffness, neurogenic pain, and central nervous system dysfunction [5–7]. hEDS patients with chronic pain may also experience fatigue, anxiety, depression, and reduced quality of life [7–10]. There is currently no curative treatment for hEDS, and the most common form of treatment is using analgesics (e.g., opioids) [2, 5, 7, 11]. Given the complications of pain medications such as tolerance [12], substance abuse [13], and persistent pain, despite medication [2], integrating complementary strategies to manage pain could be part of a comprehensive multidisciplinary treatment approach.

Several theories have been proposed to explain the mechanism(s) of chronic pain. Melzack and Wall's gate control theory (GCT) involves a bottom-up approach which posits that stimulation of peripheral nerves by nonnoxious stimuli can impede the transmission of painful stimuli [14]. Melzack later developed the neuromatrix (NM) theory of pain, a top-down model in which a collection of central nervous system components determines whether afferent noxious stimuli can be translated into pain perception [15]. Under the NM theory, sensory, cognitive, and affective aspects influence pain perception.

Rhythmic sensory stimulation (RSS) is defined as the stimulation of the senses in a periodic manner within a range of low frequencies. Music plus low-frequency sound delivered through a vibroacoustic device is a form of RSS and has demonstrated utility in managing pain [16, 17]. This intervention is based on two forms of rhythmic sensation: (1) sound captured by the auditory system and (2) vibrations captured by the somatosensory system. Although the mechanisms underpinning RSS for pain management are not well understood, theoretical grounds for utilizing RSS for treating pain in hEDS include positive cognitive/affective effects from music on brain function [18–21], neural entrainment of rhythmic stimuli [22–24], and sensory inhibition of pain via vibration [14, 25].

Although research using RSS has been conducted within an array of conditions, little research has focused on chronic pain in hEDS. In a previous single-case study of a patient with hEDS [26], we found an improvement in pain interference scores after using RSS via a vibroacoustic device for four weeks. In addition, the study found an improvement in bowel function, musculoskeletal tension, and energy levels. In the current study, we conducted an open-label pilot study of 15 patients with hEDS using RSS as the intervention. Based on a previous single-subject case report of an hEDS patient using the RSS device [26], it was anticipated that this device could improve pain interference scores as well as mood symptoms in a larger sample of hEDS patients. A washout period of two weeks followed the postintervention stage.

## 2. Materials and Methods

### 2.1. Participants

A total of 15 participants entered the study, and 14 completed the study. The inclusion criteria comprised the following: (1) a clinical diagnosis of hEDS by the clinical staff at Mount Sinai Hospital's Wasser Pain Management Centre and Medical Genetics Department or a completed and signed referral form by health care practitioners indicating diagnosis; (2) joint hypermobility; (3) confirmed joint dislocations; (4) a Beighton score of 5 or more out of 9; (5) ongoing chronic pain of at least 6 months; (6) the ability to read and write English adequately; (7) self-reported satisfactory bilateral hearing; (8) the ability to operate the supplied RSS device, the Sound Oasis VTS1000, and to pick up and return the device at the Wasser Pain Management Centre.

The exclusion criteria were as follows: (1) serious concomitant illnesses, such as malignancy and vertebral fracture; (2) active inflammatory conditions, such as spinal infection and ankylosing spondylitis; (3) history of pathological fracture; (4) pregnancy; (5) coccydynia; (6) metabolic bone disease; (7) bleeding or clotting disorder; (8) hypotension; (9) active psychiatric disorders, such as bipolar disorder, schizophrenia, severe/psychotic depression, or anxiety disorder (at the discretion of clinicians/health care practitioners instead); (10) the use of pacemakers, implanted defibrillators, or neurostimulators.

### 2.2. Design and Procedure

A one-group pre-post treatment design was implemented. There were three assessments. The first assessment served as a baseline before study initiation and utilized four self-reported measures, including the Brief Pain Inventory, Short Form (BPI-SF) [27]; the Profile of Mood States, Short Form (POMS-SF) [28]; the Short Form 36 Health Survey (SF-36) [29]; and the Center for Epidemiologic Studies Depression Scale (CES-D) [30]. Following 4 weeks of treatment, the second assessment was performed with the same four questionnaires plus a Patient Global Impression of Change (PGI-C) and a 30-minute semistructured interview. After the two-week washout period, the final assessment included the same five questionnaires and a 30-minute semistructured interview. The primary endpoints were changes in scores from baseline to the end of active treatment in the BPI-SF, POMS-SF, SF-36, CES-D, and PGI-C. Permission was obtained to use each questionnaire. A short summary of each of these measures are as follows:

1. BPI-SF: the brief pain inventory assesses the severity of pain and the impact of pain on daily functions. Although first made for cancer patients, it is reliable and valid for assessing noncancer-related pain [31]. Scores for questions are rated out of 10, with greater scores indicating worse conditions. Relief is measured as a percentage, with a greater percentage indicating better relief from pain.
2. POMS-SF: the profile of mood states is used to measure psychological distress. A five-point scale ranging from “not al all” to “extremely” is administered, and a number from 0 to 4 is associated with this scale in a direction depending on the type of questions. Greater scores are associated with worse conditions. Reliability and validity for the POMS have been conducted [32].
3. SF-36: the short form healthy survey measures the quality of health and is commonly used in health economics to calculate cost-effectiveness of a health treatment. The SF-26 consists of eight scaled scores that are weighted sums of a section of questions. Each scale is converted into a 0–100 point value in which a lower score indicates more disability and a higher score indicates less disability. Reliability and validity for the SF-36 have been conducted [33].
4. CES-D: this is a brief self-report to measure depressive symptoms. The questionnaire consists of 20 questions, scores from 0 to 3, totaling 0–60 points. Higher scores indicate greater depressive symptoms. Reliability and validity for the CES-D have been conducted [34].
5. PGI-C: PGI-C consists of one item that measures the change in patient health. The range is 0–7 with a score of 1–3 indicating improvement, 4 being no improvement, and 5–7 being worse after the treatment. Reliability and validity for the PGI-C have been conducted [35].

Secondary endpoints included changes in scores from time of treatment termination to the final assessment following the washout period. Qualitative analysis was conducted on the responses from the semistructured interviews to explore patients' experience and perceived effectiveness of the RSS system for managing pain and mood. Open-ended questions from semistructured interviews included the following questions:

1. Can you please tell me about your experience with using the RSS system?
2. Have you started any new treatments to help manage your pain since you began this study 4 weeks ago? (probe: type of treatment, duration of treatment, and effectiveness of treatment)
3. Have you changed your medication regime since you began this study 4 weeks ago? (probe: reduced or increased dose, added medication, removed medication, and switched medications)
4. If you have changed your medication regime, why did you do so? (probe: change in prescription, side effects, difference in pain, mood, or sleep levels)
5. Did you experience any negative side effects using the RSS stimulation system? (probe: if yes, what were the most bothersome side effects)
6. Did you notice any positive effects/changes that you feel are a result of using the RSS system? (probe: improvement in pain levels or sleep, changes in behaviour, attitude, coping methods, emotions, and relationships)
7. What did you dislike about using the RSS stimulation system, if anything? (probe: inconvenience, frequency or duration of use, vibrations, music, and comfort of chair)
8. Would you choose to use the RSS system in the future? (probe: if yes, how often)
9. On a scale of 0–10 with 0 representing not helpful at all and 10 representing extremely helpful, how helpful do you feel the RSS system was in helping you manage the severity of your pain?
10. On a scale of 0–10 with 0 representing not helpful at all and 10 representing extremely helpful, how helpful do you feel the RSS system was in improving your mood?
11. On a scale of 0–10 with 0 representing not helpful at all and 10 representing extremely helpful, how helpful do you feel the RSS system was in helping you manage daily activity levels?

To analyze and present this qualitative data, all answers were compiled and categorized into common phrases represented by 1-E2 words, counted, and then presented in bar graphs as a percentage of the total number of participant responses for each question of interest.

During this 4-week study, patients were given an RSS device (Sound Oasis VTS1000) which they self-administered daily for 30 minutes, five days per week. The patients were instructed to select a time of day to use the RSS device; however, it was recommended that participants use the RSS device in the morning and use it consistently at the same time. The patients were given a diary in order to record their usage and the presence or absence of side effects.

Participants were instructed to place the device on a bed or chair, turn the device on, and choose the “Energize therapy session” program that is supplied on this commercially available device. “Energize” comprises of three tracks (41 Hz to 73 Hz with 41 Hz dominant; 36 Hz to 61 Hz with 41 Hz dominant; and 36 Hz to 65 Hz with 55 Hz dominant). The music uses the bass, guitar, piano, and digital keyboard and features mono and binaural high alpha and beta entrainment. A somatosensory vibration generated by an audible low-frequency sound wave can be felt from the shoulders to the low-back, and relaxing but not “ambient” instrumental music can be heard prompting the participant to begin timing their 30-minute session. Patients were instructed to turn the low-frequency sound driven vibration intensity to 15 and the volume level of the music to 1 or 2. However, participants were informed that if discomfort was experienced due to these settings, they were to adjust the vibration and volume level to alleviate the discomfort. Participants were asked to note any deviations to the recommended procedure in the diary.

All patients consented to the study after being provided a clear description of the study and their rights of participation, withdrawal, and confidentiality. The study was conducted in accordance with the principles set out in the Declaration of Helsinki [36] and approved by a Toronto Academic Hospital Research Ethics Board.

### 2.3. Data Analysis

Descriptive statistics including percentages, means, and standard deviations were used to report demographic data such as age, gender, pain location, and pain duration. Data were analyzed by comparing baseline, posttreatment, and washout measurements using repeated measures ANOVA analysis. Interview answers were compiled, and common keywords or phrases were derived from each answer, counted, and then presented as a percentage of total participants. Outcomes were analyzed using SPSS for Windows, version 22, with 5% as the significance threshold.

## 3. Results

### 3.1. Patient Demographic Characteristics

A total of 15 patients with hEDS were recruited for participation, of which 14 completed the intervention and poststudy interviews. The average age was 35.8 years (SD = 13.2 years), ranging from 23 to 59 years. All patients were female and reported the presence of a concomitant condition such as depression, anxiety, or chronic pain conditions. More than half of the patients were either married or in a relationship. The demographic and clinical characteristics have been described in Table 1.

### 3.2. Initial Findings

**Figure 1:**
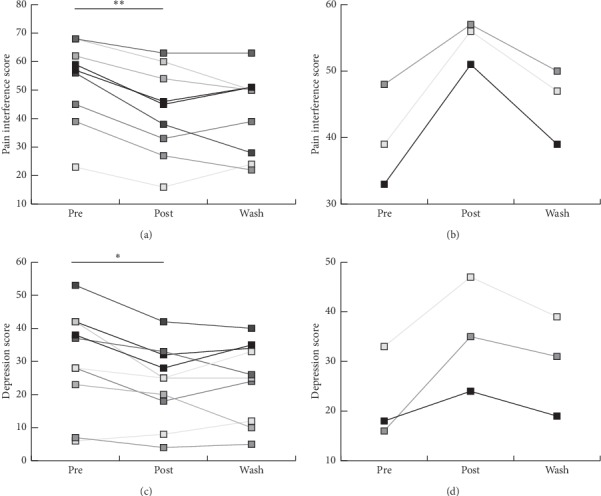
Subject-by-subject analysis of pain interference and mood in response to the intervention. (a) Line graph depicting the subgroup of patients that demonstrated improvements in BPI from preintervention to postintervention. (b) Subgroup of patients that demonstrated adverse BPI from preintervention to postintervention. (c) Subgroup analysis of patients that demonstrated improvements in CES-D from preintervention to postintervention. (d) Subgroup of patients that demonstrated adverse CES-D from preintervention to postintervention. ^*∗*^*p* < 0.05.

Posttreatment improvements in both pain interference and depression were seen in 11 of the 15 patients (73%) (Figures 1(a) and 1(c)). During the washout period, 4 of these patients (36%) reported an increase in pain interference, whereas 6 of these patients (55%) reported continued improvements. Depression symptoms generally became worse during the postwashout period with only 2 patients (18%) reporting improvements in mood. Average scores for all patients showed improvements in pain severity, pain interference, and depression from pre-post treatment but did not reach significance (*p* < 0.05, Table 2). A closer look at the data of each participant reveals that 3 of the 15 hEDS patients (20%) experienced worse outcomes in pain interference and depression symptoms after treatment, which improved after stopping the treatment (Figures 1(c) and 1(d)).

**Figure 2:**
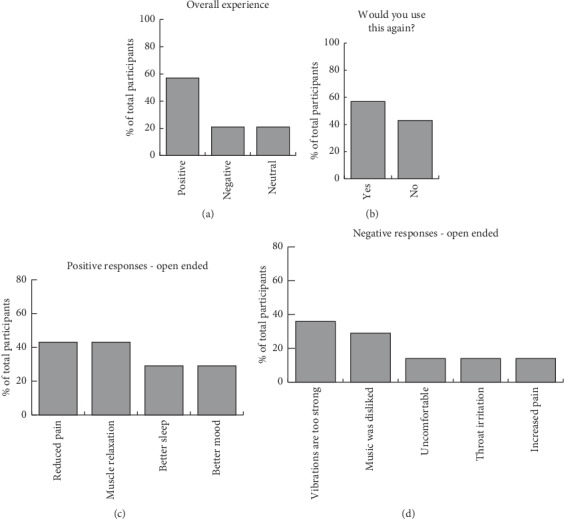
Summary of poststudy interview. (a) Overall experience of patients. (b) Response to use the intervention again. (c) Common answers to the open-ended question about positive effects. (d) Common answers to the open-ended question about negative effects.

Qualitative analysis of poststudy interviews indicated that 57% of patients reported the device to have a positive effect on their quality of life, with 21% reporting a neutral effect and 21% reporting a negative effect (Figure 2(a)). Open-ended questions suggested effects of the device on pain, muscle relaxation, sleep, and mood. Although patients were notified that they could adjust the intensity of vibrations to a comfortable level, some patients reported that the vibrations were too strong (Figures 2(c) and 2(d)). It is possible that they did not follow the RSS device adjustment instructions.

### 3.3. Clinically Meaningful Change in Pain and Depression

**Figure 3:**
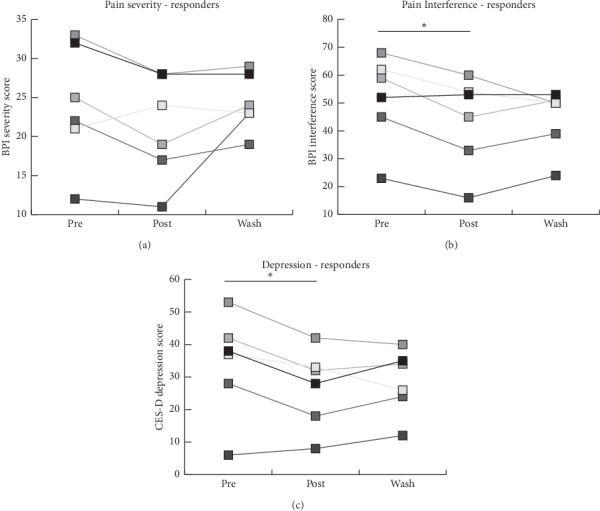
Analysis of effects of the intervention on pain interference and mood for responders. (a) Line graph depicting BPI-severity scores in patient responders at all time points in response to the intervention. (b) BPI-interference scores in patient responders in response to the intervention. (c) CES-D scores in patient responders in response to the intervention. ^*∗*^*p* < 0.05.

The analysis of individual data points along with poststudy interviews indicated that the experience of the RSS device varied among hEDS patients, with a small subset of patients reporting their symptoms worsening. Therefore, we were interested in determining the minimal clinically important difference (MCID) to report how many patients experienced a meaningful change after treatment. Following the procedure outlined in a review on MCID, the PGI-C scale was used to determine the MCID (a score of “3” or better) [37]. Of the 14 patients that completed the study, 6 participants (43%) were classified as “responders” to the device while 8 participants (57%) were classified as “nonresponders.” Mean age of the responders was found to be significantly different from the mean age of nonresponders (46.67 ± 13.2 years old for responders vs. 29.63 ± 9.1 for nonresponders, *p* < 0.05). Data analysis on responders indicated a significant improvement in pain interference and depression symptoms but not pain severity from pretreatment to posttreatment, with *p* < 0.05 (Table 3). Analysis of individual data points indicated that half of the responders continued improving in pain interference during the washout period (Figure 3(b)), whereas only 33% of these responders continued improving in depression symptoms during washout (Figure 3(c)).

### 3.4. Examining Medical Conditions in Responders and Nonresponders

**Figure 4:**
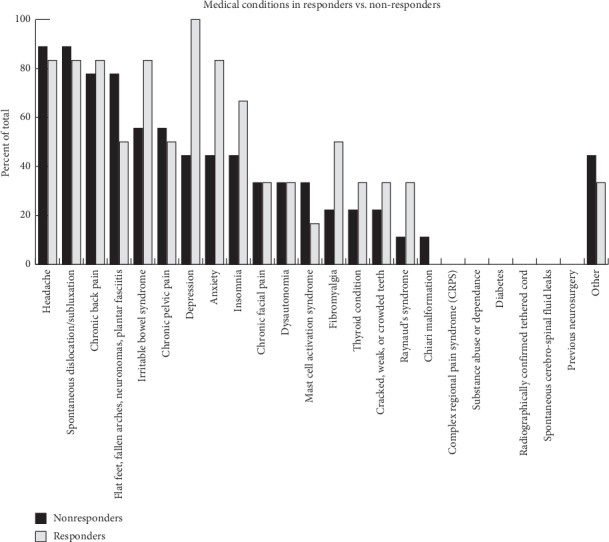
Histogram depicting the percent of total responders or nonresponders that reported having each medical condition. Nonresponders are represented in black bars, and responders are in grey. The *y*-axis represents the number of participants with each condition as a percentage of total participants. Bolded conditions represent those we considered different between responders and nonresponders.

To study the difference between responders and nonresponders in more detail, an analysis of the prevalence of concurrent medical conditions was conducted and this yielded a potentially important observation (Figure 4). Medical conditions with at least a 10 percent greater prevalence among responders than nonresponders were considered as possible candidates for screening for nonresponders. Responders had a high prevalence of depression, anxiety, insomnia, irritable bowel syndrome, and fibromyalgia.

## 4. Discussion

The present study investigated the effects of rhythmic sensory stimulation on pain and mood symptoms of hEDS patients. Our findings revealed that 43% of hEDS reported benefit from using RSS in their symptoms. The proportion of hEDS responders to this RSS treatment is similar to the proportion of fibromyalgia responders (40%) in a previous study that used a similar protocol [38]. The mean age of responders was significantly higher than the mean age for nonresponders. It is possible that older patients have a greater disease severity and more complications and are thus more likely to be responders. To study the difference between responders and nonresponders in more detail, an analysis of the prevalence of concurrent medical conditions was conducted and yielded a potentially important observation (Figure 4). Responders had a high prevalence of depression, anxiety, insomnia, irritable bowel syndrome, and fibromyalgia. These findings are consistent with observations from our previous case study. Therefore, these concurrent medical conditions could potentially serve as a selection tool to determine likely candidates among hEDS patients that may benefit from using this device.

Consistent with the initial case report, we found that pain interference is significantly improved (i.e., reduced) in responders without significant changes in pain severity. Furthermore, poststudy interviews reveal that one of the common positive benefits of the device was muscle relaxation. This suggests that the vibrotactile sensation produced by the RSS device could have improved mobility-related pain and functionality, which has been demonstrated in previous studies using low-frequency stimulation [39–41]. Mechanical stimulation of the Pacinian corpuscle, a subcutaneous mechanoreceptor sensitive to vibrations of 60 Hz to 600 Hz, is thought to produce this kind of effect [16]. Furthermore, low-frequency vibrations have been shown to produce other physiological responses that include changes in breathing rate, metabolic rate, blood pressure, fatigue, headache, nausea, and tension [42–45].

We also found significant improvements in depression symptoms in responders after using the intervention. Improvements seen in mood symptoms may be due to improvements seen in pain interference, as this increases the types of activities that patients can engage in and thus improves quality of life. However, the cognitive and affective effects of music also play a major role, increasing dopamine and serotonin levels in the brain and improving mood symptoms more directly [18, 19]. A previous study using the same intervention on patients with major depressive disorder found an improvement in depressive symptoms such as sleep quality and anhedonia after music-based RSS for five weeks [46]. The effects of music therapy on improving depression and anxiety are well documented [47].

An additional consideration is the evidence for aberrant neural oscillations that have been associated with the sensation of chronic pain [48, 49], depression [50], and poor sleep quality [51, 52]. In examining the role of rhythmic oscillatory coherence, clinical and animal studies suggest that neurogenic pain is linked to consistent abnormal activity between the thalamus and cortical areas that manifest as abnormal thalamocortical rhythms [53–58]. This phenomenon, termed thalamocortical dysrhythmia, forms the basis for a model of neurogenic pain, which is prevalent in 68% of hEDS patients [59]. Any stimuli that can normalize these aberrant neural oscillations are purported to potentially reverse or reduce the associated symptoms of chronic pain. RSS has been shown to have such an effect of synchronizing neural oscillations, and this phenomenon has been termed as entrainment in the neuroscience literature [23, 24]. The entrainment of brain rhythms to reduce chronic pain may be one of the mechanisms responsible for the benefits observed following use of RSS and must be investigated further.

## 5. Conclusion

Auditory and vibrotactile RSS can be an additional tool to reduce hEDS symptoms including pain, mood, sleep, and potentially bowel movements. hEDS patients that report these particular symptoms may be more likely to benefit from 40 Hz of auditory and vibrotactile, while a small percentage of patients may find it discomforting. Further research is necessary to elucidate the mechanisms underpinning the physical and psychological effects of RSS.

## Figures and Tables

**Table 1:** Patient demographics.

	hEDS patients (*n* = 15)	

Age	35.8 ± 13.2	
Sex	100% F	
Ethnicity	93.3% Caucasian 6.7% undisclosed	
Marital status	8 (53.3%) single	
	2 (13.3%) common law	
	3 (20.0%) married	
	2 (13.3%) divorced	
*Medical conditions*		*n* (%)
	Headache	13 (86.7%)
	Spontaneous dislocations/subluxations	13 (86.7%)
	Chronic back pain	12 (80.0%)
	Depression	10 (66.7%)
	Irritable bowel syndrome	10 (66.7%)
	Flat feet, fallen arches, neuromas, and plantar fasciitis	10 (66.7%)
	Anxiety	9 (60.0%)
	Chronic pelvic pain	8 (53.3%)
	Insomnia	8 (53.3%)
	Chronic facial pain	5 (33.3%)
	Fibromyalgia	5 (33.3%)
	Dysautonomia	5 (33.3%)
	Mast cell activation syndrome	4 (26.7%)
	Thyroid condition	4 (26.7%)
	Cracked, weak, or crowded teeth	4 (26.7%)
	Raynaud's syndrome	3 (20.0%)
	Chiari malformation	1 (6.7%)
	Other^*∗*^	6 (40.0%)

^*∗*^Other includes the number of participants that reported at least one medical condition not on the original list of medical conditions. There are no reported cases of the following medical conditions: complex regional pain syndrome, substance abuse or dependence, diabetes, radiographically confirmed tethered cord, spontaneous cerebrospinal fluid leaks, or previous neurosurgery.

**Table 2:** Comparison of mean scores across time for all patients (*n* = 15).

Outcome measure	Preintervention	Postintervention	Washout	*p* value
BPI-severity	27.15 ± 6.8	25.5 ± 7.3	26.5 ± 4.5	0.293
BPI-interference	49.9 ± 13.7	46.1 ± 14.0	43.6 ± 12.4	0.089
CES-D	28.5 ± 14.2	26.2 ± 12.2	25.6 ± 11.3	0.458
POMS_total	49.23 ± 25.56	50.08 ± 30.00	44.46 ± 22.81	0.483
SF_overall	126.92 ± 94.90	151.92 ± 89.83	144.23 ± 77.83	0.146

Analysis was done by repeated measures ANOVA. Numbers represent mean and standard deviation, *M* (±SD). No significant findings for POMS-SF or SF-36. ^*∗*^*p* < 0.05; ^*∗∗*^*p* < 0.01.

**Table 3:** Comparison of mean scores across time for all responders (*n* = 6).

Outcome measure	Preintervention	Postintervention	Washout	*p* value	Effect size (partial *η*^2^)
BPI-severity	24.2 ± 7.8	21.2 ± 6.7	24.3 ± 3.7	0.234	0.252
BPI-interference	51.5 ± 16	43.5 ± 16.4	44.5 ± 11.2	0.028^*∗*^	0.510
CES-D	34.0 ± 15.9	26.8 ± 12.1	28.5 ± 10.0	0.031^*∗*^	0.499
POMS_total	51.83 ± 27.06	45.33 ± 28.72	43.67 ± 20.20	0.31	0.211
SF_general	141.67 ± 106.85	162.50 ± 102.16	150.00 ± 75.83	0.44	0.151

Analysis was done by repeated measures ANOVA. Numbers represent mean and standard deviation. No significant findings for POMS-SF or SF-36. ^*∗*^*p* < 0.05.

## Data Availability

The data used to support the findings of this study are included within the article.

## References

[1] Castori M., Camerota F., Celletti C. (2010). Natural history and manifestations of the hypermobility type Ehlers-Danlos syndrome: a pilot study on 21 patients. *American Journal of Medical Genetics Part A*.

[2] Voermans N. C., Knoop H., Bleijenberg G., van Engelen B. G. (2010). Pain in Ehlers-Danlos syndrome is common, severe, and associated with functional impairment. *Journal of Pain and Symptom Management*.

[3] Malfait F. (2017). The 2017 international classification of the Ehlers-Danlos syndromes. *merican Journal of Medical Genetics Part C: Seminars in Medical Genetics*.

[4] Bulbena A., Gago J., Pailhez G., Sperry L., Fullana M. A., Vilarroya O. (2011). Joint hypermobility syndrome is a risk factor trait for anxiety disorders: a 15-year follow-up cohort study. *General Hospital Psychiatry*.

[5] Castori M., Morlino S., Celletti C. (2012). Management of pain and fatigue in the joint hypermobility syndrome (a.k.a. Ehlers-Danlos syndrome, hypermobility type): principles and proposal for a multidisciplinary approach. *American Journal of Medical Genetics Part A*.

[6] Hsu L. (2012). Ehlers-Danlos syndrome and chronic pain. *Journal of Pain & Palliative Care Pharmacotherapy*.

[7] Sacheti A., Szemere J., Bernstein B., Tafas T., Schechter N., Tsipouras P. (1997). Chronic pain is a manifestation of the Ehlers-Danlos syndrome. *Journal of Pain and Symptom Management*.

[8] Berglund B., Nordström G., Lützén K. (2000). Living a restricted life with Ehlers-Danlos syndrome (EDS). *International Journal of Nursing Studies*.

[9] Lumley M. A., Jordan M., Rubenstein R., Tsipouras P., Evans M. I. (1994). Psychosocial functioning in the Ehlers-Danlos syndrome. *American Journal of Medical Genetics*.

[10] Savasta S., Merli P., Ruggieri M., Bianchi L., Spartà M. V. (2011). Ehlers-Danlos syndrome and neurological features: a review. *Child’s Nervous System*.

[11] Rombaut L., Malfait F., De Wandele I. (2011). Medication, surgery, and physiotherapy among patients with the hypermobility type of Ehlers-Danlos syndrome. *Archives of Physical Medicine and Rehabilitation*.

[12] Zenz M., Strumpf M., Tryba M. (1992). Long-term oral opioid therapy in patients with chronic nonmalignant pain. *Journal of Pain and Symptom Management*.

[13] Passik S. D. (2009). Issues in long-term opioid therapy: unmet needs, risks, and solutions. *Mayo Clinic Proceedings*.

[14] Melzack R., Wall P. D. (1967). Pain mechanisms: a new theory. *Survey of Anesthesiology, *.

[15] Melzack R. (2001). Pain and the neuromatrix in the brain. *Journal of Dental Education*.

[16] Boyd-Brewer C., McCaffrey R. (2004). Vibroacoustic sound therapy improves pain management and more. *Holistic Nursing Practice*.

[17] Naghdi L., Ahonen H., Macario P., Bartel L. (2015). The effect of low-frequency sound stimulation on patients with fibromyalgia: a clinical study. *Pain Research and Management*.

[18] Salimpoor V. N., Benovoy M., Larcher K., Dagher A., Zatorre R. J. (2011). Anatomically distinct dopamine release during anticipation and experience of peak emotion to music. *Nature Neuroscience*.

[19] Erkkilä J., Punkanen M., Fachner J. (2011). Individual music therapy for depression: randomised controlled trial. *British Journal of Psychiatry*.

[20] Khalfa S., Bella S. D., Roy M., Peretz I., Lupien S. J. (2003). Effects of relaxing music on salivary cortisol level after psychological stress. *Annals of the New York Academy of Sciences*.

[21] Mitchell L. A., MacDonald R. A. R., Knussen C., Serpell M. G. (2007). A survey investigation of the effects of music listening on chronic pain. *Psychology of Music*.

[22] Huang T. L., Charyton C. (2008). A comprehensive review of the psychological effects of brainwave entrainment. *Database of Abstracts of Reviews of Effects (DARE): Quality-Assessed Reviews*.

[23] Thut G., Schyns P. G., Gross J. (2011). Entrainment of perceptually relevant brain oscillations by non-invasive rhythmic stimulation of the human brain. *Frontiers in Psychology*.

[24] Calderone D. J., Lakatos P., Butler P. D., Castellanos F. X. (2014). Entrainment of neural oscillations as a modifiable substrate of attention. *Trends in Cognitive Sciences*.

[25] Sufka K. J., Price D. D. (2002). Gate control theory reconsidered. *Brain and Mind*.

[26] Picard L., Bartel L., Gordon A. (2018). Vibroacoustic therapy for Ehlers-Danlos syndrome. *Annals of Clinical case reports*.

[27] Cleeland C. S., Ryan K. M. (1994). Pain assessment: global use of the brief pain inventory. *Annals, Academy of Medicine, Singapore*.

[28] Curran S. L., Andrykowski M. A., Studts J. L. (1995). Short form of the profile of mood states (POMS-SF): psychometric information. *Psychological Assessment*.

[29] Ware J. E., Sherbourne C. D. (1992). The MOS 36-ltem short-form health survey (SF-36). *Medical Care*.

[30] Radloff L. S. (1977). The CES-D scale. *Applied Psychological Measurement*.

[31] Keller S., Bann C. M., Dodd S. L., Schein J., Mendoza T. R., Cleeland C. S. (2004). Validity of the brief pain inventory for use in documenting the outcomes of patients with noncancer pain. *The Clinical Journal of Pain*.

[32] Gibson S. J. (1997). The measurement of mood states in older adults. *The Journals of Gerontology Series B: Psychological Sciences and Social Sciences*.

[33] Jenkinson C., Wright L., Coulter A. (1994). Criterion validity and reliability of the SF-36 in a population sample. *Quality of Life Research*.

[34] Cosco T. D., Prina M., Stubbs B., Wu Y.-T. (2017). Reliability and validity of the center for epidemiologic studies depression scale in a population-based cohort of middle-aged U.S. Adults. *Journal of Nursing Measurement*.

[35] Rampakakis E., Ste-Marie P. A., Sampalis J. S., Karellis A., Shir Y., Fitzcharles M.-A. (2015). Extended report: real-life assessment of the validity of patient global impression of change in fibromyalgia. *RMD Open*.

[36] World Medical Association (2013). World medical association declaration of Helsinki. *JAMA*.

[37] Copay A. G., Subach B. R., Glassman S. D., Polly D. W., Schuler T. C. (2007). Understanding the minimum clinically important difference: a review of concepts and methods. *Spine Journal*.

[38] Janzen T. B., Paneduro D., Picard L., Gordon A., Bartel L. R. (2019). A parallel randomized controlled trial examining the effects of rhythmic sensory stimulation on fibromyalgia symptoms. *PLoS One*.

[39] Karkkainen M., Mitsui J. (2006). The effects of sound based vibration treatment on the human mind and body: the physioacoustic method. *Journal of International Society of Life Information Science*.

[40] Cerciello S., Rossi S., Visonà E., Corona K., Oliva F. (2019). Clinical applications of vibration therapy in orthopaedic practice. *Muscles, Ligaments and Tendons Journal*.

[41] Wigram T. (1997). The effect of vibroacoustic therapy compared with music and movement based physiotherapy on multiply handicapped patients with high muscle tone and spasticity. *Collected Work: Music Vibration and Health*.

[42] Boyd-Brewer C. (2003). Vibroacoustic therapy: sound vibrations in medicine. *Alternative and Complementary Therapies*.

[43] Grocke D., Wigram T. (2007). *Vibroacoustic Therapy in Receptive Music Therapy: Techniques and Clinical Applications for Music Therapy Clinicians, Educators and Students*.

[44] Patrick G. (1999). The effects of vibroacoustic music on symptom reduction. *IEEE Engineering in Medicine and Biology Magazine*.

[45] Skille O. (1989). VibroAcoustic therapy. *Music Therapy*.

[46] Janzen T. B., Al Shirawi M. I., Rotzinger S., Kennedy S. H., Bartel L. (2019). A pilot study investigating the effect of music-based intervention on depression and anhedonia. *Frontiers in Psychology*.

[47] Maratos A., Gold C., Maratos A. (2003). Music therapy for depression. *The Cochrane Database of Systematic Reviews*.

[48] Llinás R., Ribary U., Jeanmonod D. (2001). Thalamocortical dysrhythmia I. functional and imaging aspects. *Thalamus and Related Systems*.

[49] Fu B., Wen S.-n., Wang B., Wang K., Zhang J.-y., Liu S. J. (2018). Acute and chronic pain affects local field potential of the medial prefrontal cortex in different band neural oscillations. *Molecular Pain*.

[50] Coan J. A., Allen J. J. B. (2004). Frontal EEG asymmetry as a moderator and mediator of emotion. *Biological Psychology*.

[51] Li N., Wang Y., Wang M., Liu H. (2008). Effects of sleep deprivation on gamma oscillation of waking human EEG. *Progress in Natural Science*.

[52] Gerdes L., Gerdes P., Lee S. W., Tegeler C. H. (2013). HIRREM™: a noninvasive, allostatic methodology for relaxation and auto-calibration of neural oscillations. *Brain and Behavior*.

[53] Jeanmonod D., Magnin M., Morel A. (1993). Thalamus and neurogenic pain. *NeuroReport*.

[54] Jeanmonod D., Magnin M., Morel A. (1996). Low-threshold calcium spike bursts in the human thalamus. *Brain*.

[55] Gerke M. B., Duggan A. W., Xu L., Siddall P. J. (2003). Thalamic neuronal activity in rats with mechanical allodynia following contusive spinal cord injury. *Neuroscience*.

[56] Llinas R. R., Ribary U., Jeanmonod D., Kronberg E., Mitra P. P. (1999). Thalamocortical dysrhythmia: a neurological and neuropsychiatric syndrome characterized by magnetoencephalography. *Proceedings of the National Academy of Sciences*.

[57] Stern J., Jeanmonod D., Sarnthein J. (2006). Persistent EEG overactivation in the cortical pain matrix of neurogenic pain patients. *NeuroImage*.

[58] Walton K. D., Llinás R. R. (2010). *Central Pain as a Thalamocortical Dysrhythmia: A Thalamic Efference Disconnection?*.

[59] Zhou Z., Rewari A., Shanthanna H. (2018). Management of chronic pain in Ehlers-Danlos syndrome. *Medicine*.

